# Electron paramagnetic resonance detection of superoxide in a murine model of acute lung injury

**DOI:** 10.1007/s44352-025-00014-1

**Published:** 2025-08-28

**Authors:** Hanan B. Elajaili, Nathan Dee, Tanden Hovey, Autumn Canny, Georgina Amassah, Janelle Posey, George A. Rinard, Joseph P. Y. Kao, Sandra S. Eaton, Gareth R. Eaton, Eva S. Nozik

**Affiliations:** 1 Cardiovascular Pulmonary Research Laboratories and Pediatric Critical Care Medicine, University of Colorado Anschutz Medical Campus, 12700 E. 19 Ave., Aurora, CO 80045 USA; 2 Department of Chemistry and Biochemistry, University of Denver, Denver, CO USA; 3 Department of Physiology, Center for Biomedical Engineering & Technology, University of Maryland School of Medicine, Baltimore, MD USA

**Keywords:** Acute lung injury, Superoxide, Mitochondria, Spin probe, EC-SOD, Blood

## Abstract

Superoxide (O_2_^·−^) production in an acute lung injury (ALI) murine model was detected by electron paramagnetic resonance (EPR) spectroscopy and imaging. Lung injury was induced in wild-type (WT) mice and transgenic (Tg) mice with lung-specific EC-SOD overexpression by lipopolysaccharide (LPS) administered intraperitoneally (IP) at a dose of 10 mg/kg. At 24 h after LPS treatment, mice were treated intraperitoneally and subcutaneously with the cyclic hydroxylamine probe, CMH, for superoxide measurements in the blood, or via intratracheal delivery (IT) with the cyclic hydroxylamine probes, CPH or DCP-AM-H, for lung cellular and mitochondrial superoxide detection. Blood was drawn one hour after CMH probe administration, while lungs were harvested five minutes following the administration of CPH or DCP-AM-H. Superoxide measurements in the blood by EPR were performed at X-band (~ 9.5 GHz). EPR images of isolated lungs were obtained by rapid-scan EPR at L-band (1 GHz). Inflammatory cell count, protein, and cell count in bronchoalveolar lavage fluid (BALF) were used to evaluate systemic inflammation and lung injury, respectively. Increased circulating neutrophils and monocytes indicate LPS-induced systemic inflammation. LPS-induced ALI was evidenced by increased alveolar protein and inflammatory cell count. In WT mice LPS increased superoxide in blood and increased lung cellular and mitochondrial superoxide, measured by EPR. In Tg mice with increased lung EC-SOD, blood superoxide increased; however, lung cellular and mitochondrial superoxide did not increase with LPS. These results show that EPR spectroscopy and imaging of excised lungs can detect superoxide production in a model of ALI and differentiate between cellular and mitochondrial superoxide. This provides essential new information as we showed that changes in lung superoxide does not always correlate with changes in blood superoxide levels. This is a significant step toward the ultimate goal of establishing a protocol for real-time monitoring of lung redox status in vivo, enabling disease risk stratification and guiding clinical research.

**Supplementary Information:**

The online version contains supplementary material available at 10.1007/s44352-025-00014-1.

## Introduction

Recent studies on acute respiratory distress syndrome (ARDS) highlight the importance of identifying subphenoypes of patients (hypo-inflammatory and hyper-inflammatory) that are associated with their different responses to therapies [1–4]. Despite many studies aimed at finding treatments, the mortality rate is still high and the significant long-term effects on survivors are becoming increasingly clear [5].

Inflammation contributes to the increased production of reactive oxygen species (ROS), leading to oxidative stress, a hallmark of ARDS [6]. Despite the involvement of ROS in the pathogenesis of ARDS, antioxidant treatments trials have not been proven effective in reducing the mortality of this disease [7–13]. A major contributing factor is that the lung redox status was not directly measured in patients. Oxidative stress measures rely on end point products accumulation in plasma and have been used as surrogates for ROS detection in the lung [14, 15]. Direct quantification of ROS production within the lung may be necessary for disease risk stratification and guiding clinical research, supporting the need to advance technologies to address this gap.

Electron paramagnetic resonance (EPR) spectroscopy is a vital analytical technique in redox biology. It is the “gold standard” in the detection, identification, and quantification of free radicals. Because most endogenous free radicals are highly reactive and damaging, biochemical mechanisms have evolved to detoxify such radicals. Consequently, the steady-state concentrations of these radical species are extremely low. Therefore it is essentially impossible to observe endogenous radical species directly in real time, in vivo. For example, steady-state intracellular superoxide concentration is in the nanomolar range. Thus, even if the superoxide EPR spectrum could be recorded at room temperature, the radical concentration would still be below the EPR detection limit. For this reason, spin traps are commonly used for detecting and quantifying superoxide [16, 17]. These probes react with the short-lived free radicals, including labile radical ROS, to produce more stable (long-lived) radicals that can be measured and quantified by EPR. Cyclic hydroxylamines constitute one class of spin traps. These probes react rapidly with superoxide to produce a more stable nitroxide radical, whose distinctive spectrum enables detection and quantification by EPR spectroscopy. The signal detected by EPR is proportional to the concentration of nitroxide radicals, each of which has one unpaired electron, which is commonly referred to as a “spin” in EPR parlance. The nitroxide signal is proportional to the amount of superoxide trapped during the time of the experiment (minus a decrement caused by bio-reduction of the nitroxide), and is used to determine changes in superoxide production. Probes that can permeate cell membranes and accumulate selectively in organelles (e.g., mitochondria, cytoplasm, etc.) permit comparison of O_2_^·–^ in various subcellular compartments and are promising in advancing EPR spectroscopy and imaging applications.

Monitoring changes in superoxide in vivo is possible with the injection of the spin traps in live mice [18, 19]. We are therefore developing novel EPR imaging technologies along with specialized molecular probes to track in vivo lung superoxide in an ALI mouse model. In a rapid-scan EPR experiment the magnetic field is scanned through resonance at kHz frequencies, which permits extensive signal averaging and provides substantial improvements in signal-to-noise that enable detection of signals that are weaker than can be studied by conventional EPR [20]. We have developed a spin-probe dosing strategy to monitor superoxide in LPS-treated WT and SOD-deficient mice based on EPR spectroscopy at X-band (~ 9.5 GHz) [18]. Because of the strong absorption of X-band microwaves by water-laden samples, these studies were limited to 8–10 mg of excised tissue or 50 μL of fluid. To study intact lungs or living animals, EPR experiments need to be performed at lower frequencies where energy absorption is decreased and the depth of penetration into the tissue is greater [21]. We have shown that the EPR spectra of radicals produced by the reaction between spin traps and superoxide can be detected and imaged at L-band (1 GHz) [19]. We now describe physiology in the ALI model that can be studied by L-band EPR spectroscopy and imaging of radicals produced by reaction with superoxide.

## Materials and methods

### Mouse model

Animal studies were approved by the University of Colorado Denver (Aurora, CO) Institutional Animal Care and Use Committee (IACUC). We examined C57BL/6J (WT) mice and a heterozygous mouse strain with lung-specific overexpression of EC-SOD on the C57BL/6J mouse background which were bred at the University of Colorado Denver. The sod3 transgene is driven by the surfactant protein C promoter in type II alveolar epithelial cells [22]. The EC-SOD Tg mice have been previously shown to express 2.5–4 times more lung EC-SOD and are protected against lung injury [23–32]. Mice are genotyped to identify Tg mice and WT littermates.

### Injury model and sample collection

Lung injury was induced as previously described [18]. Briefly, mice received a single dose (10 mg/kg) of lipopolysaccharide (LPS) (*E. coli* O55; Sigma) intraperitoneally (IP). We evaluated mice 24 h after LPS or vehicle (PBS) treatment. EPR probes were administered to mice deeply anesthetized with 1.5% isoflurane. Heparin-coated 21-gauge needles were used for blood collection. Mice were euthanized with CO_2_ and cervical dislocation. The chest cavity was opened and after flushing with 5 mL of cold PBS via the right ventricle, the lungs were harvested for EPR measurements. The lungs were lavaged for bronchiolavage fluid (BALF) collection by slowly instilling and withdrawing 1 mL of ice-cold PBS 3 times; this was repeated for 4 additional 1-mL aliquots. The collected five aliquots were centrifuged at 700 ×*g* for 7 min, and sedimented cells were resuspended 400 µL PBS.

### Delivery of spin probes

Three cyclic hydroxylamine probes were used in this study: 1-hydroxy-3-methoxycarbonyl-2,2,5,5-tetramethylpyrrolidine (CMH) (ENZO), 1-hydroxy-3-carboxy-2,2,5,5-tetramethylpyrrolidine hydrochloride (CPH) (ENZO), and 4-acetoxymethoxycarbonyl-1-hydroxy-2,2,5,5-tetramethylpyrrolidine-3-carboxylic acid (DCP-AM-H) (gift of Sergey Dikalov) [33]. 24 h after LPS administration, CMH, CPH or DCP-AM-H probes were delivered to mice. Stock solutions of probes were prepared in Krebs–Henseleit buffer (KHB) containing 5 μM sodium diethyldithiocarbamate and 25 μM deferoxamine mesylate salt (Sigma Aldrich). To eliminate dissolved O_2_, the solution was purged with N_2_ for 30 min. The dosing strategy was selected based on our published protocol [18]. Briefly, an intraperitoneal (IP) bolus of 90 µL CMH (18 mM stock) was administered, followed immediately by a 135 µL subcutaneous (SQ) dose of the same concentration, with an additional SQ dose given 30 min later. The IP dose rapidly establishes an adequate circulating concentration of the probe, while the SQ doses, which are absorbed more slowly, serve to maintain a more stable concentration of the probe. Our previous studies demonstrated that the SQ doses significantly enhanced the EPR signal. CPH and DCP-AM-H were administered intratracheally (IT) following anesthesia with 1–3% isoflurane. As previously described [18], 100 µL of CPH (18 mM stock) or 100 µL of DCP-AM-H (2.5 mM stock) was delivered IT. At 1 h after CMH injection, mice were placed under anesthesia, and blood was collected via right ventricular puncture using a syringe with a heparin-coated needle. Lungs were harvested 5 min after IT delivery of CPH or DCP-AM-H and kept on ice until EPR measurements. Preferential loading of mitochondria with DCP-AM-H is described in Supplemental Material.

### Evaluation of blood inflammatory cells

Inflammatory cell counts in blood were obtained using a hematologic analyzer (Heska HT5, Loveland, CO).

### Measurements in blood

Measurements of nitroxide CM· produced by reaction of spin trap CMH with superoxide in the blood were made at X-band (Bruker EMXnano). EPR analyses were performed at room temperature (RT) using a capillary tube (50 µL of blood).

### BALF protein and cell count

Total protein content in the supernatant from the BALF was measured using a BCA protein assay kit. The cell pellet was resuspended in PBS (400 µL), and a 20 µL aliquot was diluted with trypan blue (1:1) for total cell counting using a Countess II FL cell counter.

### Lung imaging and nitroxide spin quantitation

The 1 GHz spectrometer used for rapid-scan imaging is similar to the previously described 700 MHz imager [34]. The modified 1-GHz imager was used for imaging the lungs and spin quantification (the count of nitroxide molecules generated from the reaction of hydroxylamines probes with superoxide), as previously reported [19]. The spin quantitation reported in this paper was based on a non-gradient spectrum that was acquired prior to recording the spectral-spatial image.

## Data analysis

Data were analyzed with Prism software using an unpaired *t*-test. Results are presented as mean ± SEM, with statistical significance set at *p* < 0.05.

## Results

### Systemic inflammation and blood superoxide increased following IP LPS

**Fig. 1:**
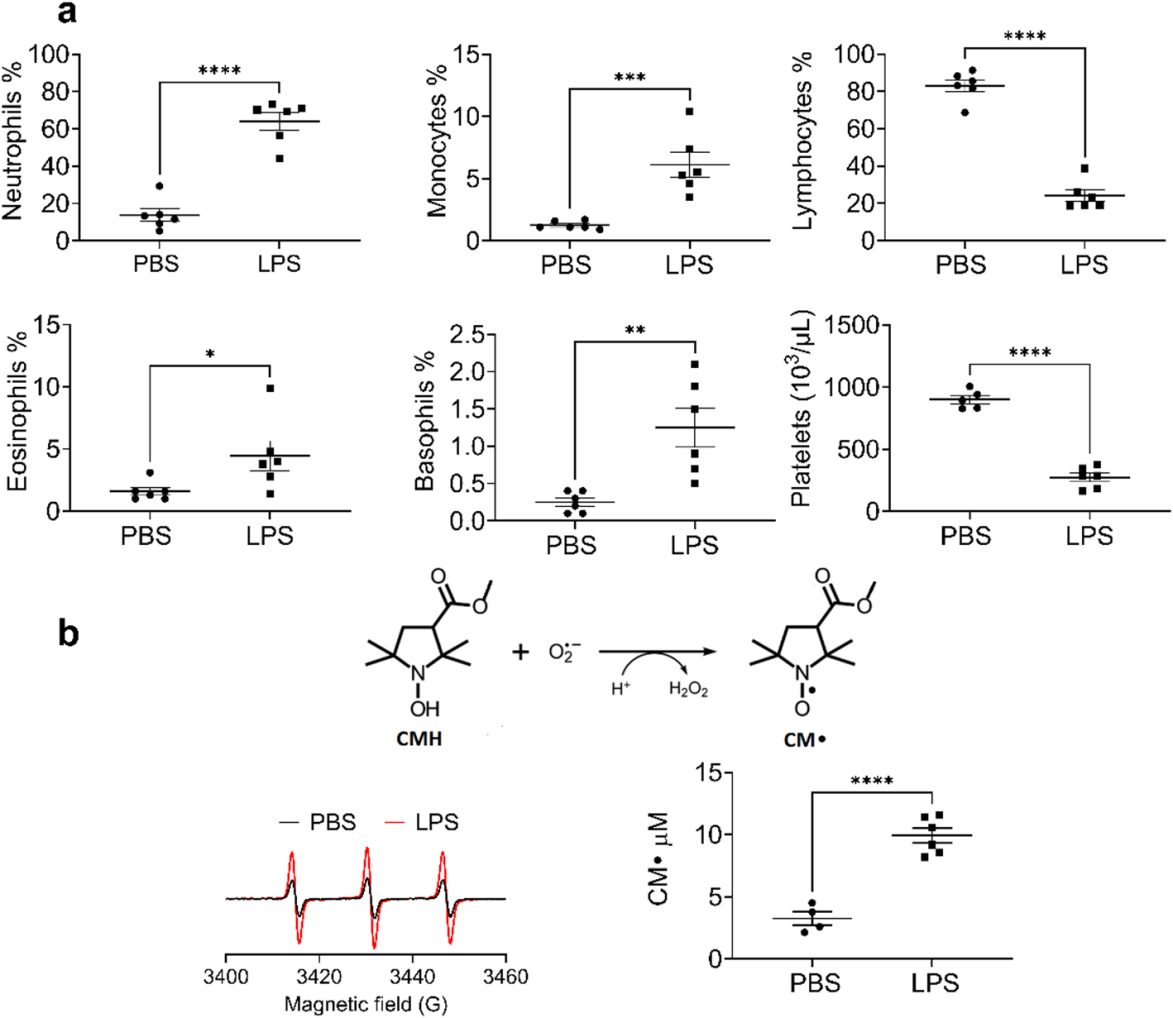
Blood superoxide and systemic inflammation are increased following IP LPS. Mice were treated with LPS (10 mg/kg; IP). **a** Cell counts in the blood. After 24 h mice were injected with CMH, with concurrent IP and SQ injection, followed by a second SQ injection 30 min later; blood was collected after a further 30 min. **b** The scheme shows the reaction of CMH with superoxide to generate the nitroxide CM·. *Left*: Representative EPR spectra of CM· in the blood of PBS-treated (black trace) and LPS-treated (red trace) mice. *Right*: CM· concentrations in blood determined by Bruker Spin Fit and spin count module. EPR measurements were conducted at X-band at room temperature. Data expressed as mean ± SEM; *p < 0.05, **p < 0.01, ***p < 0.001, ****p < 0.0001 (n = 4– 6)

We first confirmed that IP LPS induced systemic inflammation by evaluating inflammatory cell counts in the blood. Neutrophils and monocytes increased while platelets decreased after LPS exposure, consistent with sepsis. We also observed a modest but significant decrease in lymphocytes and increase in eosinophil and basophil numbers after LPS treatment (Fig. 1A). The concentration of nitroxide, CM·, generated from the reaction of CMH with superoxide in blood, was determined by Bruker Spin Fit and spin count module at X-band (Fig. 1B). LPS treatment increased blood superoxide level compared to the PBS control.

### Alveolar protein leak and BALF cell count increased following IP LPS

**Fig. 2:**
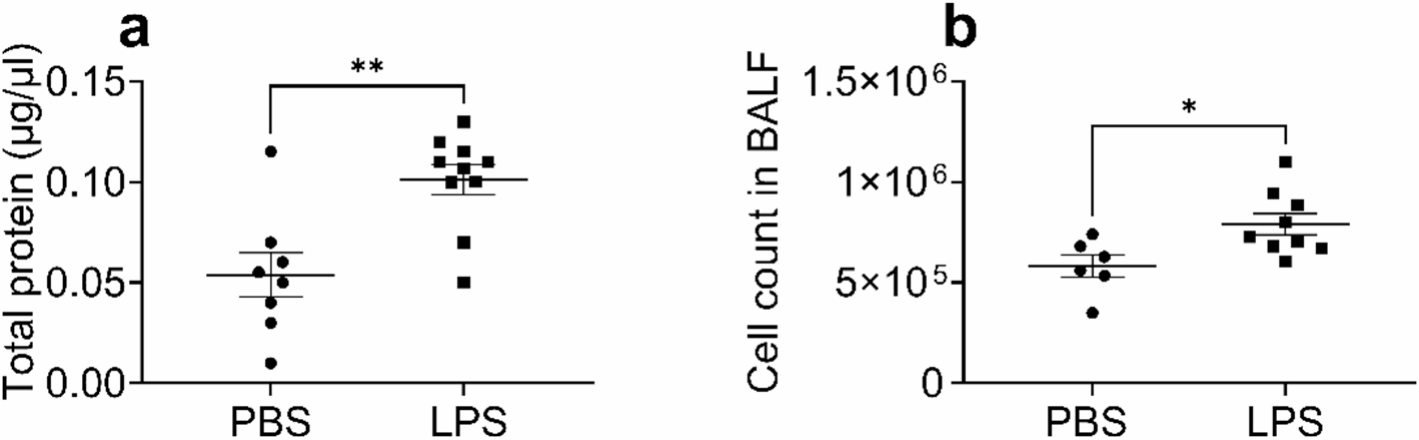
Bronchoalveolar lavage fluid (BALF) protein leak and alveolar cell count increased following IP LPS. Mice were treated with LPS (10 mg/kg, IP) and euthanized 24 h after LPS treatment. BALF was collected and analyzed for protein content and cell count. **a** Total protein concentration in BALF. **b** Total alveolar cells. Data expressed as mean ± SEM; *p < 0.05 (n = 6–10)

We then characterized the LPS-induced ALI by determining the total protein and cell count in the BALF. Protein leak in BALF reflects lung injury with disruption of the epithelial and endothelial barrier (Fig. 2A). Lung injury was also confirmed by the increase in total BALF cell count after LPS treatments (Fig. 2B).

### Increased lung cellular and mitochondrial superoxide following IP LPS by L-band EPR spectroscopy and imaging

**Fig. 3:**
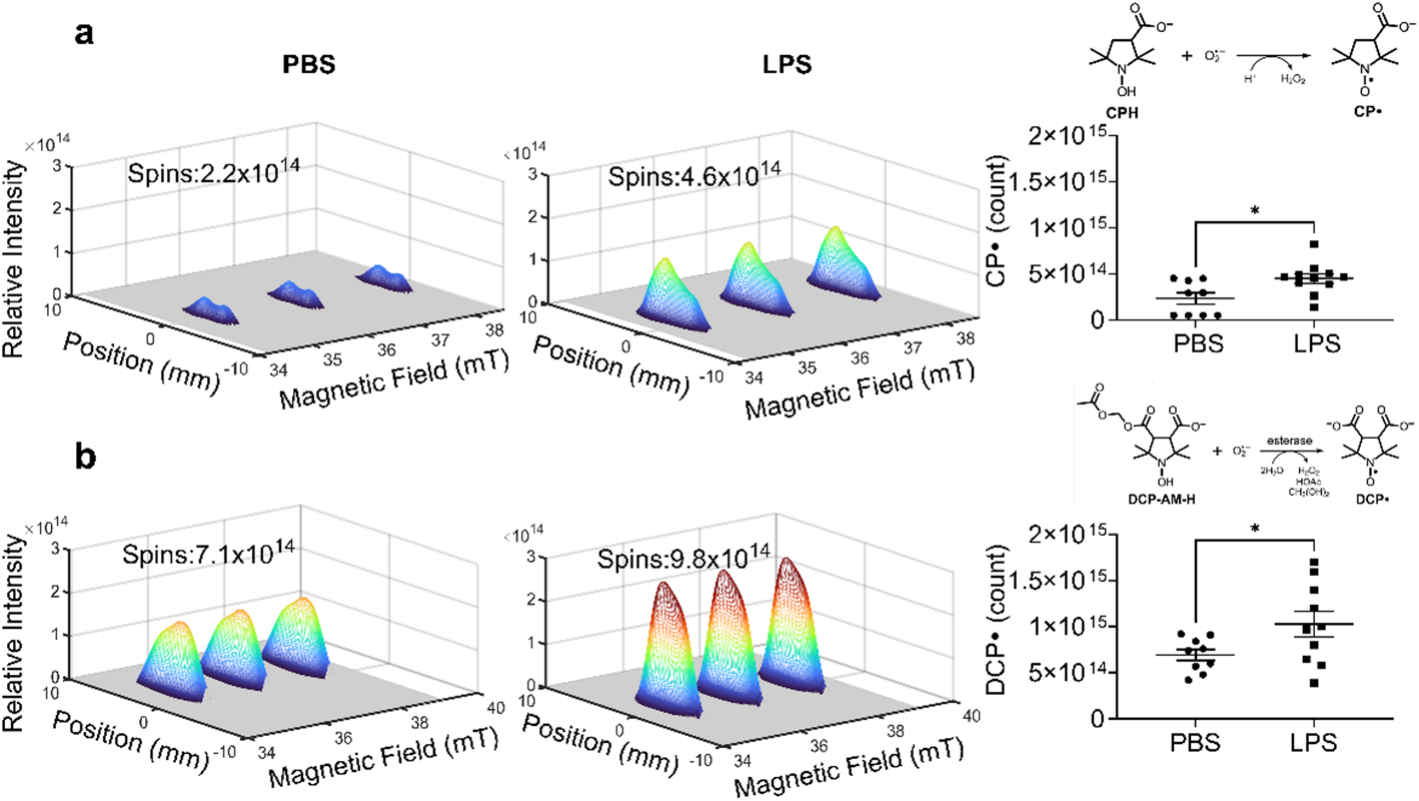
EPR imaging detects increased lung cellular and mitochondrial superoxide following IP LPS. Mice were treated with LPS (10 mg/kg, IP). 24 h after LPS treatment, EPR probes CPH or DCP-AM-H were administered intratracheally (IT), and after 5 min lungs were harvested. Spectral-spatial images show the EPR spectrum along the spatial axis that is defined by the direction of the magnetic field gradient. **a** Left two panels: the 3-line nitroxide spectrum of CP· in the lungs from PBS- and LPS-treated mice, respectively. Right panel: the number of CP· spins detected. **b** Left two panels: the 3-line nitroxide spectrum of DCP· in the lungs from PBS- and LPS-treated mice, respectively. Right panel: the number of DCP· spins detected. Data expressed as mean ± SEM; *p < 0.05 (n = 8–11)

We then tested if EPR spectroscopy and imaging can detect differences in cellular and mitochondrial superoxide and differentiate between injured and uninjured lungs. The two lungs were placed side-by-side in an EPR tube (8 mm OD and 7 mm ID) that was positioned in the magnet with the magnetic field gradient perpendicular to the tube axis. The spectral-spatial image (Fig. 3) displays the 3-line nitroxide EPR spectrum as a function of position along the direction of the gradient. No other EPR signals were observed. Fitting of lineshapes in the spectral dimension removes background noise. The variation in spectral intensity along the spatial axis is determined by the geometry of the lungs in the tube, which is maximum near the center of the tube. The resolution of the image was not high enough to distinguish signals from the two closely-spaced lungs. An increase in cellular superoxide after LPS treatment was detected using the CPH probe (Fig. 3A). Using the DCP-AM-H probe we also detected an increase in mitochondrial superoxide (Fig. 3B). The spin count of CP· and DCP· in the spectra was obtained using the procedure previously described [19]. The observation of EPR signals in the excised lungs that are strong enough to image, and the expectation of similar signal strengths in vivo, suggest the feasibility of in vivo imaging.

### Tg mice selectively overexpressing lung EC-SOD show increased blood superoxide but unchanged lung cellular or mitochondrial superoxide

**Fig. 4:**
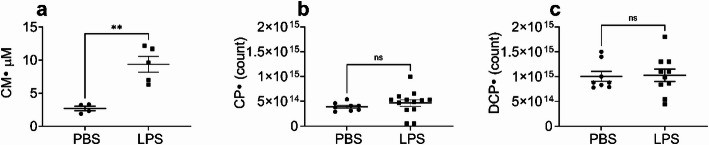
In Tg mice selectively overexpressing lung EC-SOD, IP LPS increased blood superoxide but did not increase either lung cellular or mitochondrial superoxide. Tg mice were treated with LPS (10 mg/kg, IP). 24 h after LPS treatment, mice were dosed with CMH through concurrent IP and SQ injection, followed by a second SQ injection 30 min later, with blood collected after a further 30 min. For lung cellular or mitochondrial superoxide detection mice were injected with CPH or DCP-AM-H probe intratracheally (IT); 5 min thereafter lungs were harvested. **a** CM· concentration in the blood of PBS- and LPS-treated Tg mice. **b** Number of CP· spins in the lung of PBS- and LPS-treated Tg mice. **c** Number of DCP· spins in the lung of PBS- and LPS-treated Tg mice. Data expressed as mean ± SEM; *p < 0.05, ns: no significant difference (n = 4–13)

We assessed superoxide production in the blood and the lungs of Tg mice (overexpressing EC-SOD in the lung) following LPS or PBS treatment. LPS increased superoxide production in the blood of Tg mice (Fig. 4A). In contrast, LPS did not increase lung cellular and mitochondrial superoxide in Tg mice (Fig. 4B and C).

## Discussion

Improved methodologies are needed to evaluate lung redox status in ALI accurately and minimally invasively. Measurements of free radical production are particularly challenging because biologically relevant radicals are short-lived and most existing methods rely on non-specific measures of ROS (e.g., fluorescent probes) or markers of oxidative stress (e.g., lipid peroxidation, DNA oxidation, protein carbonyls) [14, 15]. We developed an EPR method to monitor superoxide production in vivo in a preclinical model of ALI. In this study, we used IP LPS to generate systemic inflammation, evidenced by increased circulating leukocytes, and ALI, as shown by elevated alveolar protein and cell count. Mice were injected with EPR probes in vivo and the nitroxide signal produced by reaction with superoxide was measured in blood and lung ex vivo 24 h following LPS. In WT mice, LPS increased superoxide in blood– an effect associated with elevated inflammatory cells– and both cellular and mitochondrial superoxide in the lung. In contrast, in Tg mice with EC-SOD overexpression in the lung, LPS still increased blood superoxide but cellular and mitochondrial superoxide no longer increased in the lung. This emphasizes that circulating superoxide levels may not reflect the redox state of the lungs, highlighting the significance of direct lung imaging.

Our long-term goal is to develop in vivo EPR imaging of lungs to evaluate lung redox status quantitatively in ARDS. This study represents a significant step forward, as we use low-frequency EPR imaging of lungs ex vivo to quantify superoxide generation in live mice. This work builds on our recent studies where we first developed a protocol to deliver EPR probes to live mice and measured the superoxide levels by standard X-band EPR spectroscopy [18]. We then extended the work to establish a protocol with low-frequency EPR, achieving an enhanced signal-to-noise ratio by using rapid-scan EPR [19]. Other investigators have also assessed superoxide levels following LPS in live mice by using different analytical techniques. HPLC detection of superoxide-specific oxidation products of dihydroethidium (DHE) has been successful across various organs following LPS treatment, but this protocol cannot be used for imaging [35]. A previous study showed the feasibility of using in vivo injection of CPH after LPS exposure, with subsequent X-band EPR measurements at liquid nitrogen temperature [36]. Our current study advances this work by using different hydroxylamine probes to measure superoxide production in intact tissues at room temperature through low frequency (L-band) EPR. This is a key step toward future L-band EPR imaging of lungs in live mice.

It is important to note that although the hydroxylamine probes are oxidized by superoxide to the corresponding nitroxides, the measured nitroxide concentrations may not exactly mirror the superoxide concentrations in situ. This is because nitroxides are not indefinitely stable in vivo. Even the pyrrolidine nitroxide derivatives used here, which are among the most stable, are susceptible to eventual bio-reduction (e.g., by ascorbate) back to the hydroxylamine, which then can be oxidized again. Such redox processes complicate the interpretation of the measured nitroxide concentration as the total superoxide produced in vivo* during the time of an experiment*. Our focus is on changes in superoxide production, as monitored by reaction with spin traps.

The rationale to develop lung EPR imaging is that measures of the redox status in circulation may not directly correlate with lung redox status. Therefore, a direct assessment of the injured lung may provide useful information to stratify patients and response to treatment. This premise is strongly supported by the data in mice overexpressing EC-SOD in the lungs, where LPS increased circulating superoxide levels but did not increase superoxide levels in the lungs. Clinical studies testing antioxidant strategies have included blood markers of oxidative stress (e.g., protein carbonyls and MDA + 4-HNE) as surrogates for lung redox status [9–13]. Unfortunately, these antioxidant trials did not improve outcomes in the populations studied. EPR spectroscopy and imaging of superoxide offers a promising approach for real-time, in vivo assessment of lung redox status, with the potential to better stratify disease risk and guide clinical studies.

In summary, this study is an important advance toward in vivo imaging of acute lung injury in live mice. Our findings underscore the fact that circulating superoxide levels may not correlate with the redox status of the lungs, emphasizing the importance of direct lung imaging. Future studies will develop the capacity to monitor lung redox status in live animals.

## Supplementary Information

Below is the link to the electronic supplementary material.

Supplementary file1.

## Data Availability

The datasets generated during and/or analysed during the current study are available from the corresponding author on reasonable request.
