# Supplementary material for: Electron paramagnetic resonance detection of superoxide in a murine model of acute lung injury
Source: Discov Imaging. 2025 Aug 28;2(1):11. doi: 10.1007/s44352-025-00014-1 (PMC12394321; doi:10.1007/s44352-025-00014-1)
Supplement: Supplementary file 1 — Supplementary file1. [file 44352_2025_14_MOESM1_ESM.pdf]

Supplementary material

Electron Paramagnetic Resonance Detection of Superoxide in a Murine Model of Acute Lung Injury

Hanan Elajaili [1], Nathan Dee [1], Tanden Hovey [2], Autumn Canny [2], Georgina Amassah [2], Janelle Posey [1], George A. Rinard [2], Joseph P. Y. Kao [3], Sandra S. Eaton [2], Gareth R. Eaton [2], Eva S. Nozik [1]

1Cardiovascular Pulmonary Research Laboratories and Pediatric Critical Care Medicine, University of Colorado Anschutz Medical Campus, Aurora, CO

2Department of Chemistry and Biochemistry, University of Denver, Denver, CO

3Center for Biomedical Engineering & Technology, and Department of Physiology, University of Maryland School of Medicine, Baltimore, MD

Estimating preferential loading of DCP-AM-H into mitochondria

We have performed additional experiments to verify that the signal arising from DCP-AM-H is primarily mitochondrial. The antibiotic Antimycin A (AA) inhibits Complex III of the mitochondrial electron transport chain, with a consequent large increase of superoxide generation in the mitochondria. We show that AA application markedly increased the EPR signal from lung tissue loaded with DCP-AM-H (Figure 1).

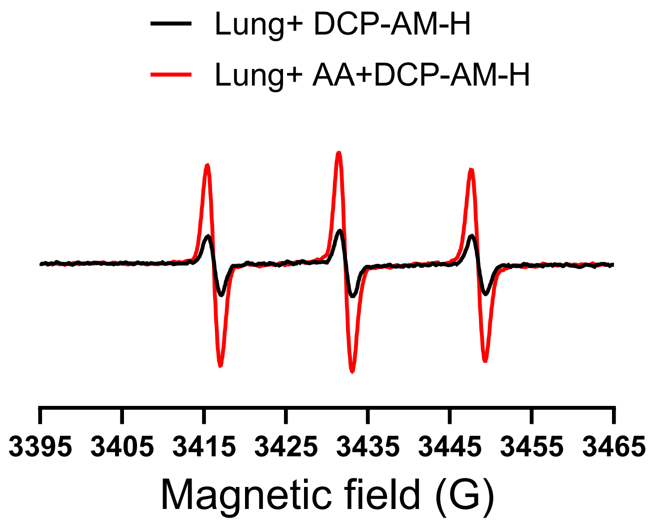

Figure 1. Lung tissue was incubated for 30 min at 37 °C with 20 μM DCP-AM-H in the absence and the presence of 20 μM Antimycin A (AA). Thereafter the nitroxide signal in the tissue was analyzed by X-band EPR spectroscopy.

Quantitation showed that AA treatment increased the nitroxide level from an average of 1.88 (± 0.17) × 10^{12} to 6.38 (± 2.77) x10^{12} spins/mg tissue – an increase by a factor of 3.40 ± 0.44. This finding can be used to calculate the extent to which DCP-AM-H preferentially localizes to mitochondria. To perform the calculation, two other data are needed: 1) The fraction of total cell volume occupied by mitochondria in lung epithelial cells is 0.063 (6.3%).[1] 2) Application of AA at micromolar concentrations increases mitochondrial superoxide production by ~10-fold.[2-4] We perform the calculation as follows.

R is the factor by which the spin trap preferentially localizes to mitochondria over the rest of the cell. Under control conditions, the whole-cell EPR signal, $S_{wc,ctrl}$, is the sum of volume-weighted contributions from the mitochondria, S_m, and the rest of the cell, S_c:

$$S_{wc,ctrl} = 0.063S_m + 0.937S_c = 0.063S_m + 0.937\frac{S_m}{R} = [0.063 + \frac{0.937}{R}]S_m$$

After AA application, the mitochondrial signal is augmented 10-fold, while the rest of the cell remains unchanged. Thus, with AA, the whole cell signal becomes

$$S_{wc,AA} = 0.063(10S_m) + 0.937\frac{S_m}{R} = [0.63 + \frac{0.937}{R}]S_m$$

Defining the ratio of $S_{wc,AA}$ to $S_{wc,ctrl}$ to be E_{AA}, we obtain

$$E_{AA} = \frac{S_{wc,AA}}{S_{wc,ctrl}} = \frac{0.63 + \frac{0.937}{R}}{0.063 + \frac{0.937}{R}} = \frac{0.63R + 0.937}{0.063R + 0.937},$$

which, upon rearrangement, gives

$$R = 1.4873\frac{E_{AA} - 1}{1 - 0.1E_{AA}}$$

Using our experimentally determined E_{AA} = 3.4*0* ± 0.44 gives *R* = 5.40 ± 1.15. Thus mitochondria are preferentially loaded by a factor of 5.4. Alternatively we can say that 5.4/(5.4+1) = 0.84, or that mitochondrial loading represents 84% of the total.

Caveats on inferring superoxide concentration from EPR measurements of superoxide spin traps

The following observations counsel caution in attempting to quantitively relate the observed nitroxide spin concentrations to the superoxide concentrations in the cells.

1) The probe takes time to enter the cells in the tissue and may not be at the same concentration everywhere in the sample even when it does enter.
2) The nitroxide production and superoxide consumption are bimolecular processes so the rates depend on both the superoxide concentration and the probe concentration. By incubating for many minutes and then assaying the endpoint, we are measuring an integrated signal that is related to the superoxide and probe concentration at every moment during that incubation.
3) The steady-state superoxide concentration in cells is not an absolute quantity; rather, it depends on the probe concentration for the following reason. If the probe concentration is low, the rate of superoxide consumption is low, and the steady-state superoxide concentration remains closer to the value it would have been without the probe reaction. If the probe concentration is high, then superoxide is consumed faster, and the steady-state superoxide

concentration will have dropped more significantly.

4) Finally, because nitroxides are not indefinitely stable *in vivo*, there could be some bioreduction of the nitroxides, which would lower the total nitroxide measured at the endpoint.

These issues make it difficult to infer intracellular superoxide concentration directly from the spin trap measurements.

1. Schierz, A.K., et al., *Distribution and volume of mitochondria in alveolar epithelial type 1 cells in infant and adult human lungs.* Histochemistry and Cell Biology, 2024. **163**(1): p. 7.
2. Xu, X. and E.A. Arriaga, *Chemical cytometry quantitates superoxide levels in the mitochondrial matrix of single myoblasts.* Analytical Chemistry, 2010. **82**(16): p. 6745-6750.
3. Dröse, S. and U. Brandt, *The mechanism of mitochondrial superoxide production by the cytochrome bc1 complex.* Journal of Biological Chemistry, 2008. **283**(31): p. 21649-21654.
4. Sundqvist, M., et al., *Elevated mitochondrial reactive oxygen species and cellular redox imbalance in human NADPH-oxidase-deficient phagocytes.* Frontiers in Immunology, 2017. **Volume 8 - 2017**.
